# Circular collimator arc versus dynamic conformal arc treatment planning for linac-based stereotactic radiosurgery of an intracranial small single lesion: a perspective of lesion asymmetry

**DOI:** 10.1186/s13014-019-1307-z

**Published:** 2019-06-03

**Authors:** Yongsook C. Lee, Yongbok Kim

**Affiliations:** Department of Radiation Oncology, The University of Arizona, Banner University Medicine North Building #2, 3838 N Campbell Avenue, Tucson, AZ 85719 USA

**Keywords:** Circular collimator arcs, Dynamic conformal arcs, Linac-based stereotactic radiosurgery, Asymmetry, Conformity index, V12Gy

## Abstract

**Background:**

Although circular collimator arcs (CCA) and dynamic conformal arcs (DCA) are commonly used linear accelerator-based treatment planning techniques for intracranial stereotactic radiosurgery (SRS) of a small single lesion, these two techniques have not been rigorously compared in terms of tumor shape. Therefore, this study compared clinical CCA plans with re-planned DCA plans using conformity index (CI) and V12Gy (volume of normal brain tissue receiving 12 Gy or higher) from a perspective of asymmetry (Asym) of planning target volume (PTV).

**Methods:**

Ninety-five clinical CCA plans delivered for a small single lesion with PTV size < 1.4 cm^3^ were selected and re-planned using DCA. PTV Asym (%) was defined and calculated from three dimensions of PTV. A pair of the 95 plans was first considered as one group without grouping and then categorized into two groups with respective to either PTV size or PTV Asym, and four groups with respect to PTV size and PTV Asym. For grouping, median values of PTV size and PTV Asym were used. A non-parametric paired test was performed for CI and V12Gy to compare CCA and DCA plans in each group.

**Results:**

Median values of PTV size and PTV Asym were 0.415 cm^3^ (range: 0.076 cm^3^–1.369 cm^3^) and 6.12% (range: 0.52–25.74%), respectively. DCA plans had a lower average CI value than CCA plans for all groups. CCA plans had a smaller average V12Gy value than DCA plans for lesions with PTV Asym ≤6.12%, while CCA and DCA plans had similar average V12Gy values for lesions with PTV Asym > 6.12%.

**Conclusions:**

The DCA technique is recommended when a lesion has PTV Asym > 6.12% regardless of PTV size. For lesions with PTV Asym ≤6.12%, a technique choice would depend on the preference of CI or V12Gy.

## Background

One of the treatment modalities for brain metastases is stereotactic radiosurgery (SRS) [1, 2]. SRS is generally intended for treating small lesions and size of lesions is usually limited to ≤4 cm [3–5]. A wide array of treatment techniques for intracranial SRS are available and such techniques include the Gamma Knife™ (Elekta AB, Stockholm, Sweden), medical linear accelerator (linac)-based systems, charged particle (proton or other heavy ions) therapy, helical TomoTherapy (Accuray Inc., Sunnyvale, CA) and the robot-assisted linac system, Cyber Knife™ (Accuray Inc., Sunnyvale, CA) [5, 6]. Of these, linac-based systems are capable of multiple delivery techniques such as circular collimator arcs (CCA), dynamic conformal arcs (DCA), intensity modulated radiation therapy and volumetric modulated arc therapy (VMAT) [5]. While CCA uses circular cones, the rest of the techniques require micro multileaf collimators (mMLCs). Treatment of multiple lesions with a single isocenter approach has been employed since VMAT was introduced and its delivery has become highly efficient [7, 8].

Despite the advent of VMAT and its efficiency, CCA and DCA are still commonly used for the treatment of a single lesion. Both techniques are relatively simple and easy to be planned and delivered for a single lesion with one isocenter. The CCA technique uses multiple noncoplanar converging arcs with tertiary circular cones and is a conventional method for linac-based SRS [5, 9]. This technique is ideally suited for a small spherically-shaped tumor [3, 10]. Treatment of an irregularly-shaped tumor is possible using CCA but it often requires multiple isocenters for a good conformity at the expense of treatment time and dose homogeneity [9–13]. On the other hand, the DCA technique uses multiple noncoplanar arcs about a single isocenter with continuously changing field shape using mMLCs [12, 14]. It is usually used for a large and/or irregularly-shaped tumor [3, 9, 10].

SRS plan quality is evaluated with various dosimetric parameters and two common parameters are conformity index (CI) and volumes of normal brain tissue receiving at least 8–12 Gy (e.g., V12Gy). CI is presented as a ratio of the total volume enclosed by the prescription isodose to the target volume [5, 15]. CI indicates a degree of conforming the prescription isodose to shape of the target and for SRS treatment, CI values below 2.0 are recommended by the Radiation Therapy Oncology Group [13]. V12Gy is defined as the volume of normal brain tissue (total brain volume minus planning target volume (PTV)) receiving 12 Gy or higher [1, 16] and is known as the most significant prognostic factor for brain radionecrosis (RN) in SRS patients [1, 17, 18]. It has been reported that the risk of symptomatic RN rapidly increases when V12Gy is greater than 5–10 cm^3^ [17–19].

Although it is known that CCA is usually suited for a small spherically-shaped tumor and DCA is for a large and/or irregularly-shaped tumor [3, 10], a comparison of these two techniques in terms of tumor shape has not been rigorously discussed in the literature. In this study, herein, CCA and DCA techniques were compared from a perspective of lesion asymmetry using the two dosimetric parameters, CI and V12Gy, for SRS of a small (volumes < 1.4 cm^3^) single lesion with a single isocenter approach.

## Methods

### Patient cohort

A total of 95 patients who received intracranial SRS treatment for a small (volume < 1.4 cm^3^) single lesion were selected for the current retrospective study. Treatments for all the selected patients were planned and delivered using CCA. For the same patient cohort, re-planning was performed using DCA. This study was approved by our institutional review board.

### Clinical circular collimator arc treatment planning

For each patient, CCA treatment planning was performed in the iPlan RT (ver. 4.5.5, Brainlab AG, Munich, Germany) treatment planning system (TPS) which supports a pencil beam algorithm with tissue inhomogeneity corrections. Before treatment planning, PTV was constructed by a 1-mm expansion from gross tumor volume delineated with assistance of magnetic resonance images. Five to six noncoplanar partial (90–160 degrees) arcs with circular cones (from 7.5 mm to 17.5 mm in diameters in 2.5 mm increments) were configured with a single isocenter. Various beam parameters such as gantry angles, couch angles, arc length, cone size and beam weighting for each arc were adjusted such that the plan met our institutional SRS planning criteria. The planning criteria include PTV coverage (COV) ≥98.3%, CI ≤1.5 if possible, V12Gy ≤3.3 cm^3^ and prescribed isodose line (IDL) between 50 and 80%. For 95 plans, a prescribed dose ranged from 16 Gy to 24 Gy with a median value of 21 Gy. Sixteen plans with a prescribed dose other than 21 Gy were retrospectively re-scaled to 21 Gy. Dose grid size was 1 mm × 1 mm × 1.5 mm.

### Dynamic conformal arc treatment planning

Ninety-five DCA plans were generated from the 95 clinical CCA plans in the same TPS. Circular cones in each CCA plan were replaced with mMLCs to create a DCA plan. Based on our institutional practice, an MLC aperture margin was set to 0 mm to achieve similar prescribed IDL to that for CCA plans. Isocenter position, gantry and couch angles, arc length for each arc and beam weighting were kept the same as for the CCA plan. Each DCA plan was adjusted by changing prescribed IDL such that PTV COV was within ±1.0% from that for the corresponding CCA plan.

### Asymmetry calculation for each PTV

Asymmetry (Asym) was defined and calculated for each PTV. Following response evaluation criteria in solid tumors 1.1 [20], the longest diameter of PTV and its perpendicular diameter on the transverse plane were measured. Then the longest dimension perpendicular to the transverse plane was measured. From the measurements, PTV Asym was computed using the following equation:

1 $$ \mathrm{Asym}\ \left(\%\right)=\frac{\left|\mathrm{a}-\mathrm{m}\right|+\left|\mathrm{b}-\mathrm{m}\right|+\left|\mathrm{c}-\mathrm{m}\right|}{4\mathrm{m}}\times 100 $$

where a, b and c are three dimensions of PTV and m is a mean value of a, b and c. The value “4” in the denominator is a normalization factor to scale Asym (%) ranging from 0% (most symmetric) to 100% (most asymmetric).

### Grouping and statistical analysis

To compare CCA and DCA plans, statistical analyses were performed for three different cases. First, without grouping, 95 clinical CCA plans were compared with re-planned DCA plans using four dosimetric parameters (COV, IDL, CI and V12Gy) (one-group analysis). Second, a pair of the 95 plans was categorized into two groups with respect to either PTV size or PTV Asym and then CCA and DCA plans in each group were compared using two dosimetric parameters (CI and V12Gy) (two-group analysis). Third, a pair of the 95 plans was categorized into four groups with respect to both PTV size and PTV Asym, and then CCA and DCA plans in each group were compared using CI and V12Gy (four-group analysis). For each group analysis, a non-parametric test (i.e., Wilcoxon matched pairs test) was run. A difference in a dosimetric parameter between CCA and DCA plans was considered statistically significant when the *p*-value was < 0.05. It is noted that CI and V12Gy were used to compare CCA and DCA plans (one-group, two-group and four-group analyses) while COV and IDL were used to generate DCA plans with similar plan quality to CCA plans.

## Results

### Two independent variables for grouping: PTV size and PTV Asym

**Fig. 1:**
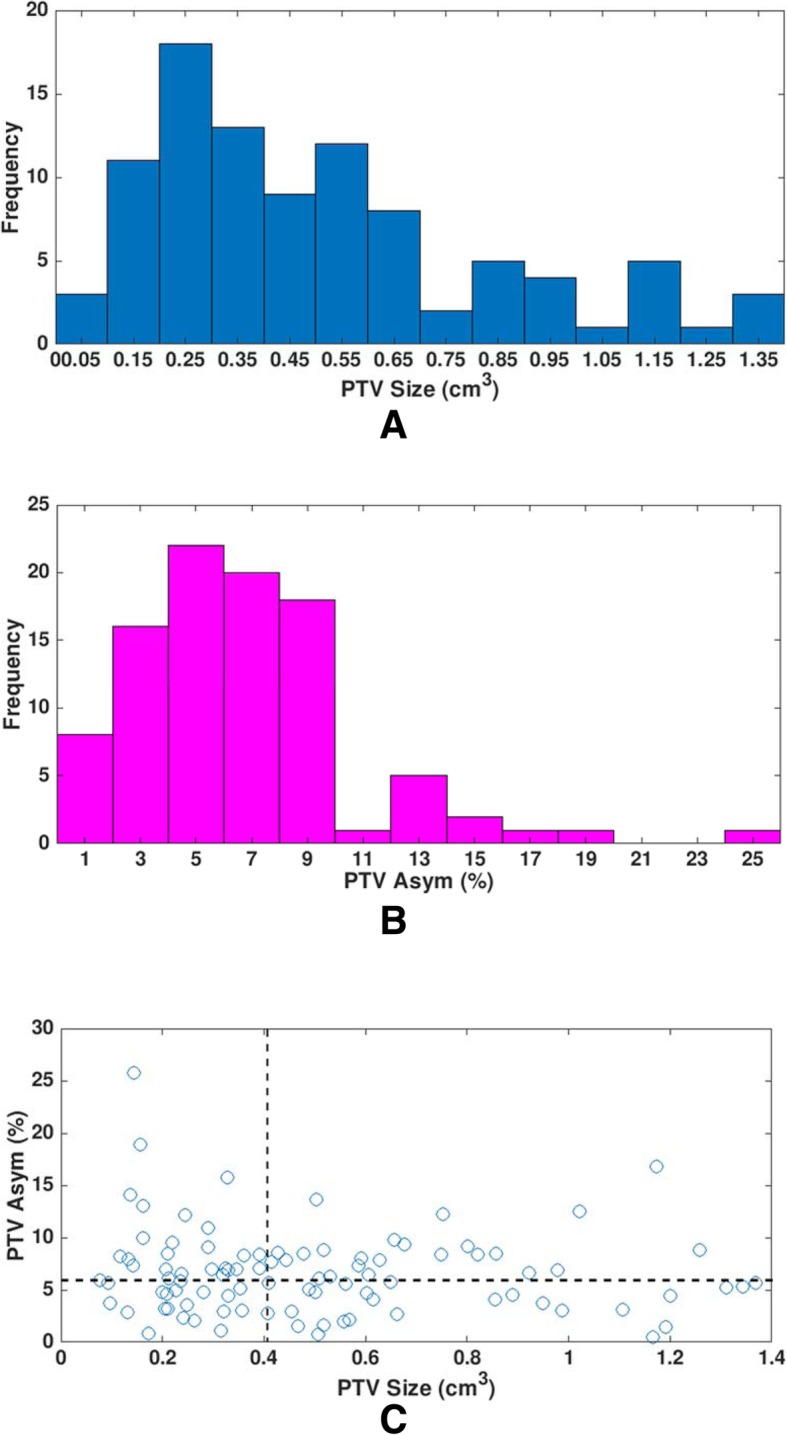
Distributions of (**a**) planning target volume (PTV) size and (**b**) PTV asymmetry (Asym), and (**c**) the plot of PTV Asym as a function of PTV size for 95 lesions. Dotted lines represent median values of PTV size (0.415 cm^3^) and PTV Asym (6.12%)

Statistics of PTV size and PTV Asym for 95 lesions is as follows. Mean ± standard deviation of PTV size is 0.505 cm^3^ ± 0.333 cm^3^ and its range (minimum - maximum) is 0.076 cm^3^–1.369 cm^3^ with a median value of 0.415 cm^3^. Those for PTV Asym are 6.64% ± 4.10 and 0.52% - 25.74% with a median value of 6.12%. As shown in Fig. 1a and b, PTV size and PTV Asym did not follow normal distributions and were skewed to low values. PTV Asym as a function of PTV size is displayed in Fig. 1c. There was no distinct trend (R^2^ = 0.011 from linear regression line) observed between PTV size and PTV Asym. The median values of PTV size and PTV Asym (dotted lines in Fig. 1c) were used to categorize a pair of the 95 plans for two-group analysis and four-group analysis.

### One-group analysis

Statistics of COV and IDL for 95 CCA and DCA plans is summarized in Table 1. COV for CCA was slightly higher than that for DCA (mean: 98.9% vs. 98.8%) and the COV difference between the two techniques was statistically significant (*p*-value = 0.0023). Mean of IDL (78.9%) for CCA was also higher than that (78.2%) for DCA and the difference in IDL was statistically significant (*p*-value = 0.0144).

**Table 1:** Comparison between circular collimator arc (CCA) and dynamic conformal arc (DCA) treatment planning for 95 lesions. Planning target volume (PTV) coverage (COV) (%), prescribed isodose line (IDL) (%), conformity index (CI), and V12Gy (cm^3^) were compared between CCA and DCA

Parameters	COV (%)		IDL (%)		CI		V12Gy (cm^3^)	
Planning technique	CCA	DCA	CCA	DCA	CCA	DCA	CCA	DCA
Minimum	98.3	98.5	73.6	73.7	1.17	1.15	0.31	0.32
Maximum	99.5	99.5	83.1	81.2	2.42	1.56	3.42	3.44
Mean	98.9	98.8	78.9	78.2	1.46	1.30	1.37	1.43
Median	99.0	98.8	79.4	78.4	1.41	1.28	1.29	1.34
Standard deviation	0.28	0.23	2.00	1.40	0.18	0.09	0.72	0.71
*p*-value	0.0023		0.0144		< 0.0001		< 0.0001	

**Fig. 2:**
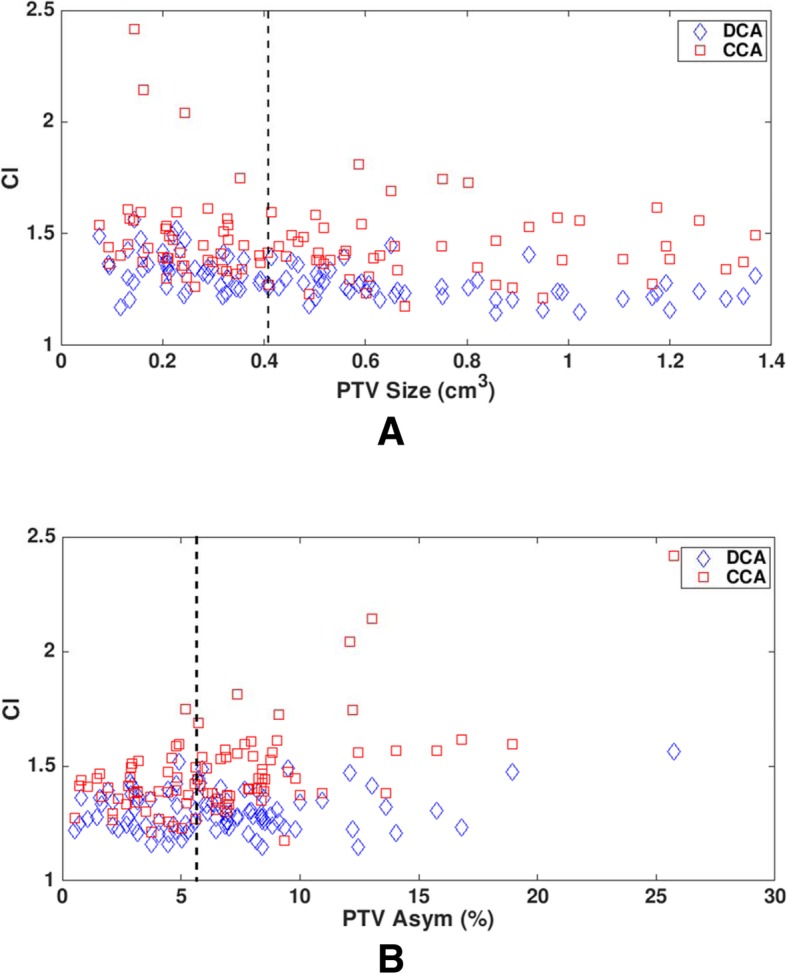
Conformity index (CI) as a function of (**a**) planning target volume (PTV) size and (**b**) PTV asymmetry (Asym) for 95 lesions. Dotted lines in (**a**) and (**b**) represent median values of PTV size (0.415 cm^3^) and PTV Asym (6.12%), respectively

CI comparison between CCA and DCA is shown in Table 1 and Fig. 2. More conformal plan was feasible with DCA than with CCA (mean: 1.30 vs. 1.46), resulting in a statistically significant difference (*p*-value < 0.0001) between the two techniques (Table 1). CI distributions as a function of PTV size showed that there was no noticeable trend (R^2^ = 0.2778 for DCA and R^2^ = 0.0256 for CCA from linear regression lines) observed between CI and PTV size (Fig. 2a). Similarly, CI distributions as a function of PTV Asym revealed that there was no dependence of CI on PTV Asym (R^2^ = 0.0334 for DCA and R^2^ = 0.3436 for CCA from linear regression lines) (Fig. 2b). As shown in Fig. 2, for most lesions, CI values were < 1.5 with DCA, satisfying our institutional SRS planning criteria better than with CCA. The upper 95% confidence interval (i.e., 97.5 percentile value) of CI for DCA was 1.49 in comparison with that (1.97) for CCA. In CCA plans, there were three lesions with CI values > 2.0. Those lesions had fairly small PTV sizes (0.144 cm^3^ - 0.244 cm^3^) and high PTV Asym values (12.1–25.7%).

**Fig. 3:**
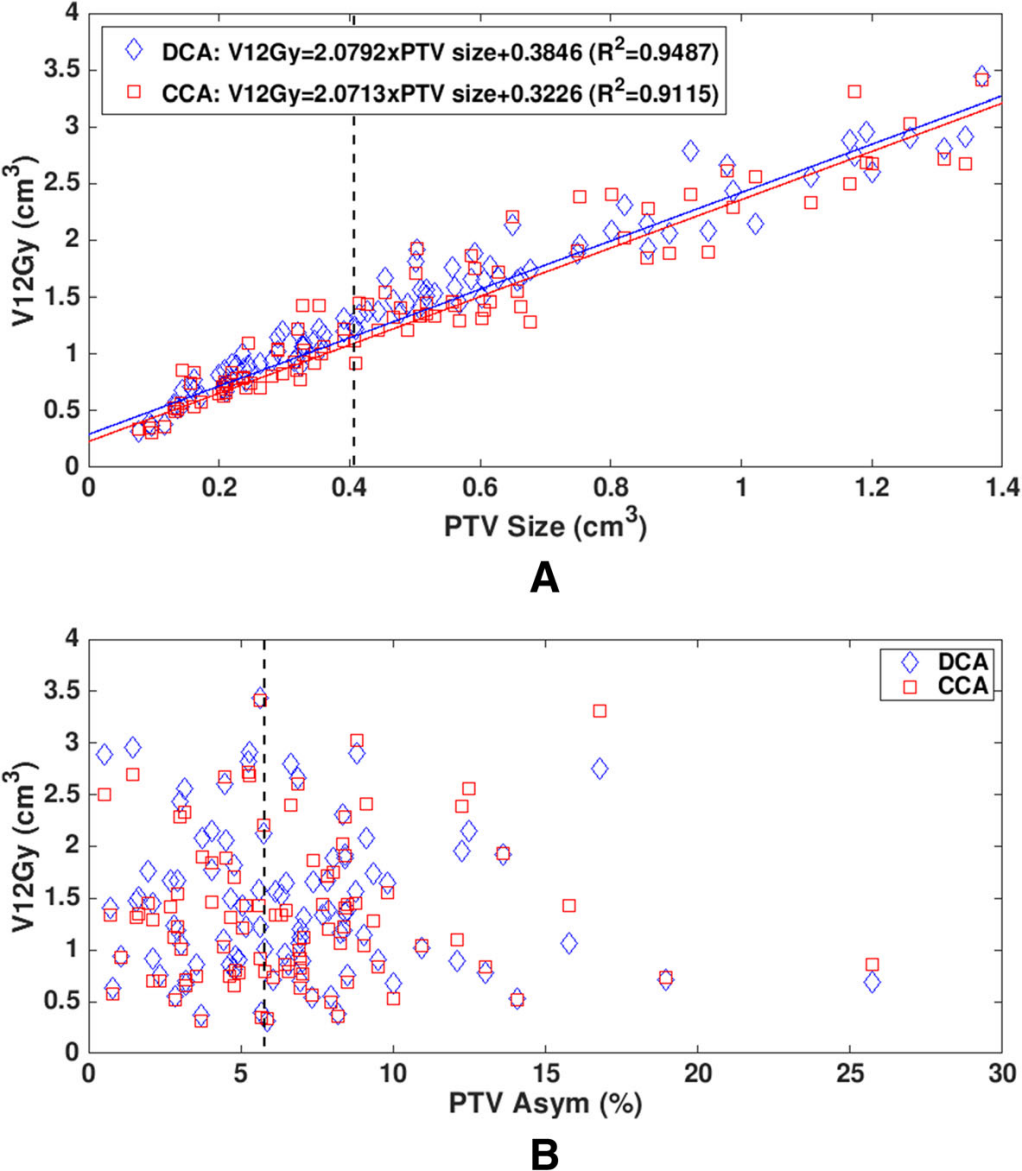
V12Gy as a function of (**a**) planning target volume (PTV) size and (**b**) PTV asymmetry (Asym) for 95 lesions. Dotted lines in (**a**) and (**b**) represent median values of PTV size (0.415 cm^3^) and PTV Asym (6.12%), respectively

V12Gy comparison between CCA and DCA is presented in Table 1 and Fig. 3. Average V12Gy (1.37 cm^3^) for CCA was smaller than that (1.43 cm^3^) for DCA and the difference was statistically significant (*p*-value < 0.0001). V12Gy had a linear relationship with PTV size for each of CCA and DCA plans (Fig. 3a). Linear regression lines are V12Gy = 2.0713 × PTV + 0.3226 for CCA plans and V12Gy = 2.0792 × PTV + 0.3846 for DCA plans, where V12Gy is the volume receiving 12 Gy or higher in cm^3^ for the prescribed dose of 21 Gy and PTV is the target volume in cm^3^. DCA plans had a better fit to the linear regression line than CCA plans (R^2^: 0.9487 vs. 0.9115). Figure 3b shows V12Gy distributions as a function of PTV Asym for 95 lesions. PTV Asym did not affect V12Gy distributions (R^2^ = 0.0088 for DCA and R^2^ = 0.0013 for CCA from linear regression lines). As shown in Fig. 3, for most lesions, CCA plans had smaller V12Gy values than DCA plans.

### Two-group analysis

#### Analysis with respect to PTV size

Table 2 shows two-group analysis with respect to PTV size. A pair of the 95 plans was divided into two groups (Group A: PTV size ≤0.415 cm^3^; Group B: PTV size > 0.415 cm^3^) using the median value (0.415 cm^3^) of PTV size. Sample sizes were 48 (50.5%) and 47 (49.5%) for Groups A and B, respectively.

**Table 2:** Two-group analysis in terms of planning target volume (PTV) size. For each group, CI and V12Gy were compared between circular collimator arc (CCA) and dynamic conformal arc (DCA) treatment planning

Group	A		B	
PTV (cm^3^)	≤0.415		> 0.415	
Sample size (%)	48 (50.5%)		47 (49.5%)	
CI comparison	CCA	DCA	CCA	DCA
Minimum	1.26	1.17	1.17	1.15
Maximum	2.42	1.56	1.81	1.45
Mean	1.49	1.34	1.43	1.26
Standard deviation	0.22	0.09	0.14	0.07
*p*-value	< 0.0001		< 0.0001	
V12Gy (cm^3^) comparison	CCA	DCA	CCA	DCA
Minimum	0.31	0.32	1.21	1.36
Maximum	1.45	1.34	3.42	3.44
Mean	0.82	0.87	1.93	2.01
Standard deviation	0.28	0.27	0.60	0.54
*p*-value	0.0017		0.0051	

For Group A, DCA plans had a lower average CI value than CCA plans (1.34 vs. 1.49). The CI difference between the two techniques was statistically significant (*p*-value < 0.0001). For Group B, similar results were observed. The average CI value (1.26) for DCA plans was lower than that (1.43) for CCA plans and the difference was statistically significant (*p*-value < 0.0001). Therefore, CI is lower in DCA plans than in CCA plans regardless of PTV size.

For Group A, CCA plans had a smaller average V12Gy value than DCA plans (0.82 cm^3^ vs. 0.87 cm^3^). There was a statistically significant difference in V12Gy between the two techniques (*p*-value = 0.0017). For Group B, average V12Gy (1.93 cm^3^) for CCA plans was also smaller than that (2.01 cm^3^) for DCA plans and the difference was statistically significant (*p*-value = 0.0051). Therefore, V12Gy for CCA plans is smaller than for DCA plans regardless of PTV size.

#### Analysis with respect to PTV Asym

Table 3 summarizes two-group analysis with respect to PTV Asym. A pair of the 95 plans was divided into two groups (Group C: PTV Asym ≤6.12%; Group D: PTV Asym > 6.12%) using the median value (6.12%) of PTV Asym. Sample sizes were 48 (50.5%) and 47 (49.5%) for Groups C and D, respectively.

**Table 3:** Two-group analysis in terms of planning target volume (PTV) asymmetry (Asym). For each group, CI and V12Gy were compared between circular collimator arc (CCA) and dynamic conformal arc (DCA) treatment planning

Group	C		D	
Asym^a^ (%)	≤6.12		> 6.12	
Sample size (%)	48 (50.5%)		47 (49.5%)	
CI comparison	CCA	DCA	CCA	DCA
Minimum	1.21	1.16	1.17	1.15
Maximum	1.75	1.52	2.42	1.56
Mean	1.41	1.30	1.52	1.30
Standard deviation	0.11	0.09	0.22	0.09
*p*-value	< 0.0001		< 0.0001	
V12Gy (cm^3^) comparison	CCA	DCA	CCA	DCA
Minimum	0.31	0.32	0.36	0.38
Maximum	3.42	3.44	3.31	2.90
Mean	1.36	1.48	1.38	1.39
Standard deviation	0.74	0.77	0.71	0.64
*p*-value	< 0.0001		0.4002	

^a^The definition of Asym (%) is in Eq. (1)

For Group C, DCA plans had a lower average CI value than CCA plans (1.30 vs. 1.41). The CI difference was statistically significant (*p*-value < 0.0001). For Group D, the average CI value (1.30) for DCA plans was also lower than that (1.52) for CCA plans and the difference was statistically significant (*p*-value < 0.0001). Therefore, CI is lower in DCA plans than in CCA plans regardless of PTV Asym.

For Group C, CCA plans had a smaller average V12Gy value than DCA plans (1.36 cm^3^ vs. 1.48 cm^3^). The V12Gy difference was statistically significant (*p*-value < 0.0001). For Group D, average V12Gy (1.38 cm^3^) for CCA plans was almost the same as that (1.39 cm^3^) for DCA plans and the difference was not statistically significant (*p*-value = 0.4002). Therefore, V12Gy is smaller in CCA plans than in DCA plans for lesions with PTV Asym ≤6.12%, while there is no difference in V12Gy between the two techniques when PTV Asym is > 6.12%.

### Four-group analysis

CI and V12Gy comparisons among four groups (Groups #1-#4) divided using median values of PTV size (0.415 cm^3^) and PTV Asym (6.12%) are presented in Table 4. Sample sizes of the four groups are 23 (24.2%), 25 (26.3%), 25 (26.3%) and 22 (23.2%) for Groups #1-#4 in order.

**Table 4:** Four-group analysis in terms of planning target volume (PTV) size and PTV asymmetry (Asym). For each group, CI and V12Gy were compared between circular collimator arc (CCA) and dynamic conformal arc (DCA) treatment planning

Group #	1		2		3		4	
PTV (cm^3^)	≤0.415		> 0.415		≤0.415		> 0.415	
Asym^a^ (%)	≤6.12		≤6.12		> 6.12		> 6.12	
Sample size (%)	23 (24.2%)		25 (26.3%)		25 (26.3%)		22 (23.2%)	
CI comparison	CCA	DCA	CCA	DCA	CCA	DCA	CCA	DCA
Minimum	1.26	1.22	1.21	1.16	1.30	1.17	1.17	1.15
Maximum	1.75	1.52	1.69	1.45	2.42	1.56	1.81	1.41
Mean	1.44	1.35	1.38	1.26	1.54	1.33	1.49	1.26
Standard deviation	0.11	0.08	0.11	0.07	0.27	0.10	0.15	0.06
*p*-value	0.0001		< 0.0001		< 0.0001		< 0.0001	
V12Gy (cm^3^) comparison	CCA	DCA	CCA	DCA	CCA	DCA	CCA	DCA
Minimum	0.31	0.32	1.21	1.41	0.36	0.38	1.21	1.36
Maximum	1.43	1.24	3.42	3.44	1.45	1.34	3.31	2.90
Mean	0.77	0.84	1.89	2.07	0.87	0.90	1.97	1.95
Standard deviation	0.27	0.27	0.61	0.60	0.28	0.26	0.59	0.47
*p*-value	0.0013		< 0.0001		0.1266		0.8486	

^a^The definition of Asym (%) is in Eq. (1)

DCA plans had lower average CI than CCA plans for all four groups. Two groups with PTV size > 0.415 cm^3^ (Groups #2 and #4) had the lowest average CI value (1.26) when DCA was used. Group #3 (PTV size ≤0.415 cm^3^ and PTV Asym > 6.12%) had the highest (1.54) when CCA was used.

For two groups with PTV Asym ≤6.12% (Group #1 and #2), CCA plans had a smaller average V12Gy value than DCA plans. On the other hand, for two groups with PTV Asym > 6.12% (Groups #3 and #4), the two techniques had similar V12Gy values (*p*-value > 0.05). In Group #3 (PTV size ≤0.415 cm^3^ and PTV Asym > 6.12%), V12Gy for CCA was slightly smaller than for DCA (0.87 cm^3^ vs. 0.90 cm^3^), whereas in Group #4 (PTV size > 0.415 cm^3^ and PTV Asym > 6.12%), V12Gy for CCA was slightly larger than for DCA (1.97 cm^3^ vs. 1.95 cm^3^). Group #1 (PTV size ≤0.415 cm^3^ and PTV Asym ≤6.12%) had the smallest average V12Gy value (0.77 cm^3^) with CCA. Group #2 (PTV size ≤0.415 cm^3^ and PTV Asym > 6.12%) had the largest (2.07 cm^3^) with DCA.

## Discussion

This study limited PTV size to < 1.4 cm^3^ in comparing CCA and DCA plans for SRS treatment. Minniti et al.’s outcome study for 310 brain metastases reported that the actuarial risk at 1 year for the development of brain RN was 0% in the first quartile (V12Gy < 3.3 cm^3^) [18]. To be conservative, our institution follows this V12Gy constraint. Our experience has shown that lesions with PTV size > 1.4 cm^3^ usually do not meet this V12Gy constraint in CCA plans. Hence, all subjects included in this study had PTV size < 1.4 cm^3^.

As shown in the Results, PTV size and PTV Asym do not affect CI in CCA and DCA plans (Fig. 2) and CI is always lower with DCA (Tables 2, 3 and 4). This would be attributed to field shaping capability of mMLCs in DCA plans. Several studies also reported that mMLCs conform to large and/or irregularly-shaped tumors better than circular cones [3, 9, 10].

**Fig. 4:**
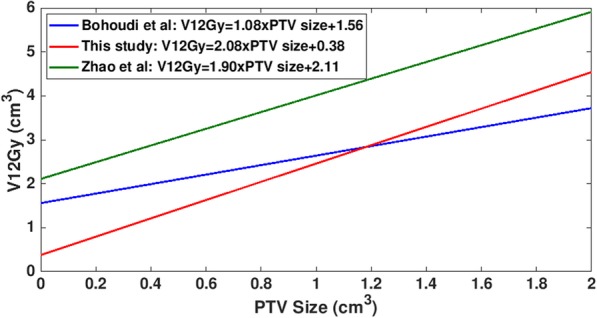
Comparison of three V12Gy prediction models for the dynamic conformal arc (DCA) technique

In this study, linear relationships between V12Gy and PTV size for CCA and DCA techniques were established (Fig. 3a). Bohoudi et al. derived a linear prediction model of V12Gy for a given prescribed dose from 30 single brain metastases with PTV size ranging from 0.14 cm^3^ to 43.4 cm^3^ treated using DCA: V12Gy = (0.12 × prescribed dose – 1.44) × PTV + (0.12 × prescribed dose – 0.96) [1]. This prediction model for a prescribed dose of 21 Gy leads to V12Gy = 1.08 × PTV + 1.56 (Fig. 4). The comparison of this model with our linear model (V12Gy = 2.08 × PTV + 0.38 for DCA) shows that Bohoudi et al.’s V12Gy values are larger than ours for PTV size < 1.2 cm^3^ (Fig. 4). This comparison is supported by Bohoudi et al.’s finding: their model slightly overestimates V12Gy for small tumors [1]. Zhao et al. also derived a linear prediction model of V12Gy (V12Gy = 1.90 × PTV + 2.11) from 22 clinical targets with volumes from 0.5 cm^3^ to 41.7 cm^3^ treated using DCA [14]. The slope (1.90) of their model is close to ours (2.08) (Fig. 4). However, their model was generated for an MLC aperture margin of 1 mm, whereas ours was obtained without a margin. As a result, their V12Gy values are larger than ours by a range of 1.4 cm^3^ to 1.7 cm^3^. Except for these models, there has been no published data on a V12Gy prediction model especially for small tumors. Therefore, the linear relationships between V12Gy and PTV size for CCA and DCA techniques obtained in this study would allow for estimation of V12Gy for PTV size < 1.4 cm^3^ and for the prescribed dose of 21 Gy.

The V12Gy comparisons between CCA and DCA shown in Tables 2, 3 and 4 demonstrated that V12Gy is smaller with CCA regardless of PTV size. However, V12Gy is smaller with CCA for lesions with PTV Asym ≤ 6.12%, while there is no difference in V12Gy between CCA and DCA techniques for lesions with PTV Asym > 6.12%. Tertiary cones used for the CCA technique increase a source to diaphragm distance in a linac, resulting in a decrease of geometric penumbra [21]. Sharper penumbra can reduce a V12Gy value in CCA plans. However, when PTV Asym becomes larger (> 6.12%), in CCA plans, both CI and V12Gy values are increased due to less flexibility of field shaping with cones. In contrast, in DCA plans, field shaping capability of mMLCs compensates for an increase of V12Gy. As a result, V12Gy becomes similar in the two techniques (1.38 cm^3^ for CCA vs. 1.39 cm^3^ for DCA) (Table 3). Therefore, lesions with PTV Asym > 6.12% can benefit from the DCA technique in maintaining similar V12Gy.

A dose gradient index (GI) is another tool to measure dose fall off outside the target and can be a useful predictor of adverse outcomes in the same way as V12Gy [22, 23]. The GI, originating from the Gamma Knife treatment of benign lesions, is defined as the ratio of the volume of half the prescription isodose to the volume of the prescription isodose [22, 23]. In our study, V12Gy was preferred to the GI for plan comparison based on our institutional practice. For 95 patients (one-group analysis), GI (mean ± standard deviation: 3.22 ± 0.40; range: 2.51–4.23) for CCA plans is lower than that (3.82 ± 0.47; 3.03–5.00) for DCA plans and the difference is statistically significant (*p*-value < 0.0001) from the Wilcoxon matched pairs test. For two-group (A vs. B or C vs. D) and four-group (1, 2, 3 vs. 4) analyses, the results are similar: GI for CCA plans is lower and the differences are statistically significant (*p*-value < 0.05).

Several studies [23–25] in the literature showed that the MLC margin between − 1.5 mm and − 0.5 mm yields the minimum GI. A margin < 0 mm would reduce V12Gy (and GI) but prescribed IDL will become lower to achieve the PTV coverage goal. Our institutional practice shows that most clinical CCA plans had prescribed IDL between 75 and 80% even though our planning criteria allow for prescribed IDL between 50 and 80%. Likewise, most clinical DCA plans had prescribed IDL between 75 and 80% and a MLC aperture margin of 0 mm was used. Therefore, based on our institutional practice, in the current study, 0 mm margin was selected to generate DCA plans which have similar PTV coverage and prescribed IDL to those for CCA plans.

Group analyses presented in this study would be useful in choosing an SRS planning technique between CCA and DCA for a small single lesion (Tables 2, 3 and 4). From the information on PTV size and PTV Asym, a better technique can be selected in terms of CI and V12Gy. For lesions with PTV Asym > 6.12% (Groups #3 and #4), the DCA technique would be better to achieve lower CI without compromising V12Gy as discussed above. For those lesions, using DCA, CI values can be improved by 0.21 for Group #3 (PTV Asym > 6.12% and PTV size ≤0.415 cm^3^) and by 0.23 for Group #4 (PTV Asym > 6.12% and PTV size > 0.415 cm^3^). For lesions with PTV Asym ≤6.12% (Groups #1 and #2), the choice of a technique would depend on the dosimetric parameter (CI or V12Gy) to which the clinician pays more attention. If lower CI is preferred, DCA would be a better option with compromised V12Gy. If smaller V12Gy is preferred, CCA should be used but CI would be increased.

## Conclusions

DCA plans have lower CI than CCA plans regardless of PTV size or PTV Asym. On the other hand, CCA plans have smaller average V12Gy for lesions with PTV Asym ≤6.12% but the two techniques have similar V12Gy when PTV Asym is larger than 6.12%. Therefore, for lesions with PTV Asym > 6.12%, regardless of PTV size, the DCA technique would be more beneficial to achieve lower CI and simultaneously, to maintain similar V12Gy compared with the CCA technique.

## Data Availability

Not applicable.

## Abbreviations

Asym: Asymmetry
CCA: Circular collimator arcs
CI: Conformity index
COV: Coverage
DCA: Dynamic conformal arcs
IDL: Isodose line
linac: Linear accelerator
mMLCs: Micro multileaf collimators
PTV: Planning target volume
RN: Radionecrosis
SRS: Stereotactic radiosurgery
TPS: Treatment planning system
V12Gy: Volume of normal brain tissue receiving 12 Gy or higher
VMAT: Volumetric modulated arc therapy
